# Cognitive training with casual video games: points to consider

**DOI:** 10.3389/fpsyg.2013.01010

**Published:** 2014-01-07

**Authors:** Pauline L. Baniqued, Michael B. Kranz, Michelle W. Voss, Hyunkyu Lee, Joshua D. Cosman, Joan Severson, Arthur F. Kramer

**Affiliations:** ^1^Department of Psychology, Beckman Institute for Advanced Science and Technology, University of Illinois at Urbana ChampaignUrbana, IL, USA; ^2^Department of Psychology, University of IowaIowa City, IA, USA; ^3^Brain Plasticity InstituteSan Francisco, CA, USA; ^4^Department of Psychology, Vanderbilt UniversityNashville, TN, USA; ^5^Digital Artefacts LLCIowa City, IA, USA

**Keywords:** attention, working memory, reasoning, fluid intelligence, video games, cognitive training, casual games, transfer of training

## Abstract

Brain training programs have proliferated in recent years, with claims that video games or computer-based tasks can broadly enhance cognitive function. However, benefits are commonly seen only in trained tasks. Assessing generalized improvement and practicality of laboratory exercises complicates interpretation and application of findings. In this study, we addressed these issues by using active control groups, training tasks that more closely resemble real-world demands and multiple tests to determine transfer of training. We examined whether casual video games can broadly improve cognition, and selected training games from a study of the relationship between game performance and cognitive abilities. A total of 209 young adults were randomized into a working memory–reasoning group, an adaptive working memory–reasoning group, an active control game group, and a no-contact control group. Before and after 15 h of training, participants completed tests of reasoning, working memory, attention, episodic memory, perceptual speed, and self-report measures of executive function, game experience, perceived improvement, knowledge of brain training research, and game play outside the laboratory. Participants improved on the training games, but transfer to untrained tasks was limited. No group showed gains in reasoning, working memory, episodic memory, or perceptual speed, but the working memory–reasoning groups improved in divided attention, with better performance in an attention-demanding game, a decreased attentional blink and smaller trail-making costs. Perceived improvements did not differ across training groups and those with low reasoning ability at baseline showed larger gains. Although there are important caveats, our study sheds light on the mixed effects in the training and transfer literature and offers a novel and potentially practical training approach. Still, more research is needed to determine the real-world benefits of computer programs such as casual games.

## INTRODUCTION

What does it mean to “train your brain”? “Brain training games” have increased in popularity over the last decade, with findings and even stronger claims that computer-based tasks of working memory and attention can broadly improve cognition (Jaeggi et al., 2008; Sternberg, 2008; Karbach and Kray, 2009; Klingberg, 2010; Morrison and Chein, 2011). However, there is often insufficient data to support these claims, with many pilot experiments^1^ and studies showing improved performance on trained tasks but limited transfer to unpracticed tasks (Willis and Schaie, 1986; Ball et al., 2002; Green and Bavelier, 2003; Willis et al., 2006; Ackerman et al., 2010; Boot et al., 2010; Owen et al., 2010; Mackey et al., 2011; Lee et al., 2012). Some training programs are plagued by replication failures (Boot et al., 2008; Owen et al., 2010; Chooi and Thompson, 2012; Redick et al., 2012; Shipstead et al., 2012; Kundu et al., 2013; Thompson et al., 2013), and methodological issues involving only single tests of transfer to cognitive abilities, placebo effects, and the lack of appropriate active control groups (Boot et al., 2011, 2013). Many programs are also costly and “games” based on laboratory tasks pose implementation concerns in terms of motivation, adherence, and task specialization.

In this study, we use a variety of casual video games, validated by their quantitative association with cognitive constructs, to train aspects of cognitive function such as reasoning ability, working memory, and attentional control. In the validation study (Baniqued et al., 2013), we used a combination of cognitive task analysis, correlational analyses, and structural equation modeling to identify casual games that were most highly associated with well-studied tasks of working memory and reasoning or fluid intelligence. Casual games are relatively easy to learn, widely and freely available on the web and on handheld devices, and can be completed in short periods of time, although they still involve a wide array of cognitive skills, complex rules, and challenging objectives. Unlike laboratory-based “games” that train cognitive abilities in more sterile or controlled paradigms, video games demand execution of skills in an integrated or more externally valid environment. For example, multitasking and working memory abilities are tapped in a game (Sushi Go Round) that involves juggling between learning and preparing different recipes correctly, ordering ingredients to keep up with demand, and cleaning the tables to make way for new customers, whereas the laboratory-based dual n-back paradigm requires participants to remember pairs of auditory and visual stimuli in sequence, with predictable order (n-back), timing, and identity of stimuli.

In addition to the richness of the game environments, the novelty and challenge from playing multiple games – akin to athletic “cross-training” (Mackey et al., 2011) may better lead to maximal engagement and gains in cognitive abilities (Green and Bavelier, 2008; Holmes et al., 2009; Schmiedek et al., 2010; Bavelier et al., 2012; Brehmer et al., 2012). The overarching goal of training endeavors is to maintain or improve everyday functioning, so programs should aim to prepare an individual for a variety of challenges. Moreover, skill acquisition research has long shown that training programs that are variable, adaptive, promote cognitive flexibility, and discourage task-specific mastery lead to greater and broader learning (Schmidt and Bjork, 1992; Kramer et al., 1995, 1999). We cannot directly evaluate these concepts in the current study, though they provide a general rationale for the approach of using multiple games to improve cognition.

Training games were selected based on a quantitative analysis of the relationship between game performance and specific cognitive abilities (Baniqued et al., 2013). In the current study, a total of 209 young adults were randomized into four groups: (1) WM-REAS 1, a group that trained on four games (one adaptive across sessions) that heavily tapped working memory and reasoning ability, (2) WM-REAS 2, another working memory–reasoning group that employed four games that were all adaptive across sessions to maximally challenge performance, (3) an active control group that trained on four games (one adaptive across sessions) that did not heavily tap working memory and reasoning, as well as a (4) no-contact control group to better assess practice effects. The WM-REAS groups played a mix of working memory and reasoning games, as validation experiments (Baniqued et al., 2013) showed that these games highly correlated with tests of reasoning and working memory, with little differentiation between the degree of correlation with the two constructs – an unsurprising finding given the integrative nature of the games and the demonstrated relationship between working memory and reasoning abilities (Carpenter et al., 1990; Colom et al., 2004; Kane et al., 2004; Unsworth and Engle, 2006; Jaeggi et al., 2008; Salthouse and Pink, 2008; Conway and Getz, 2010).

In the initial validation study, principal component analysis (PCA) of the games also showed that the WM-REAS 1 games clustered together. This further confirmed that they tapped similar skills, consistent with an *a priori* cognitive task analysis on the casual games. Moreover, structural equation modeling showed that fluid intelligence best predicted performance on most of the games, but that fluid intelligence and working memory accounted for most of the variance in the WM-REAS 1 games at 27 and 14%, respectively (Baniqued et al., 2013). Not surprisingly, correlation coefficients between WM-REAS 1 games and working memory and reasoning tasks were 0.5–0.6 at a composite level, and 0.3–0.5 at an individual task level, all significant at *p* < 0.001. Meanwhile, the active control games did not cluster together and were the least correlated with working memory and reasoning measures, with individual game by task correlations ranging from non-significant to a maximum of around 0.25. Because not all of the WM-REAS 1 games could be implemented to be adaptive across sessions (limitations due to third-party sourcing of the games), we ran a similar validation study on more games that had the ability to be adaptive across sessions. We identified those that showed comparable robust relationships with the same working memory and reasoning tasks used to evaluate WM-REAS 1 games. These additional across-session adaptive games were used for the WM-REAS 2 group (for more detail, see Supplementary Methods^2^). Given the comparable results in the second validation study, the WM-REAS 1 and WM-REAS 2 games differed mainly in their adaptive component. Three out of the four WM-REAS 1 games were not across-session adaptive and may be more susceptible to automaticity or increased reliance on task-specific mastery, and thus not maximally engage working memory and reasoning skills that can better generalize to other paradigms. That is, although we hypothesize that the WM-REAS groups would show greater improvements in cognition compared to the active and no-contact control groups, the WM-REAS 2 group may show larger gains as complex skills are continually challenged for the duration of training.

To address issues in interpreting training and transfer effects, we employed comparable training groups as mentioned above, multiple tests of each cognitive ability, and a post-experiment survey that assessed perceived improvement and inquired about game play outside of the laboratory. The inclusion of a non-WM-REAS active control group was important for assessing whether differential expectations regarding the skills tapped during training may influence performance of the transfer tasks, akin to a placebo effect (Boot et al., 2011, 2013). We also aimed to shed light on the mixed results in the cognitive training literature by discussing our results in the context of previous findings, taking into account video games and laboratory-based experiments, as well as examining individual differences that may have implications for the efficacy of game training.

To summarize, our main predictions consisted of the following: (1) WM-REAS training, given its demand on complex skills, will broadly improve cognition, (2) Individuals lower in cognitive ability (as indexed by a composite measure of reasoning tasks) will show the greatest gains from WM-REAS training, and (3) Given the integrative nature of casual games, improvement expectations will not differ between the WM-REAS and active control groups, thus making a stronger case for the utility of casual game training.

## MATERIALS AND METHODS

### PARTICIPANTS

Participants were recruited from the Champaign-Urbana community through flyers, newspaper, and online postings advertising participation in a “cognitive training study.” Applicants were first screened via email with a questionnaire that surveyed basic demographic information (e.g., sex, education, English language proficiency), and time spent playing video and board games. To mask the purpose of the game questions, these items were embedded with other lifestyle and activity questions that included the Godin Leisure-Time Exercise Questionnaire (Godin and Shephard, 1997). If not excluded based on the survey, a phone interview was conducted to check for medical and non-medical conditions that may affect neuropsychological testing. Although we focus only on the behavioral effects in this paper, we also collected brain scans for the study and thus screened for safety in a magnetic resonance imaging (MRI) environment. Eligible participants were (1) right-handed, (2) between the ages 18 and 30, (3) had normal or corrected-to-normal vision, (4) had no major medical conditions, (5) reported no non-removal metal on their body that might present a safety hazard in the MRI or affect image quality, and (6) reported playing video and board games for 3 h or less per week in the last 6 months. A total of 209 young adults completed the study (see **Table 1** for information on excluded participants and other basic demographic information). All participants signed an informed consent form approved by the University of Illinois Institutional Review Board. Upon study completion, participants were paid $15 an hour for laboratory visits. Participants who dropped out or were disqualified after the first testing session were paid $7.50 an hour. Due to the scale of the study and multitude of tasks administered, detailed procedures can be found in a supplementary document at 

**Table 1:** Demographics.

Demographics	WM-REAS 1	WM-REAS 2	Active control	No-contact
Did not complete study due to various reasons	11	12	17	18
Dropped in analysis due to video game play	10	12	9	8
Maximum analysis *N*	43	40	44	43
Male	12	11	12	12
Age	21.16 (2.25)	21.35 (2.61)	20.80 (2.10)	20.70 (2.19)
Years of education	14.78 (1.24)	15.00 (1.83)	14.67 (1.28)	14.80 (1.64)

*Shown in the first row is the number of participants excluded from analysis due to study withdrawal, non-compliance with experiment procedures, or scheduling difficulties. During the post-experiment survey, the participants reflected in the second row reported being an active game player or playing the training or testing games outside the lab. All the succeeding measures include only participants (maximum analysis *N*) not excluded based on the first two criteria. Standard deviations are shown in parentheses.*

### STUDY DESIGN

All participants underwent three cognitive testing sessions and an MRI session in a fixed session and task order (**Table 2**). Participants were randomly assigned to one of four groups: working memory and reasoning games (WM-REAS 1), adaptive working memory and reasoning games (WM-REAS 2), active control casual games that did not correlate with working memory and reasoning, or a no-contact control group (**Table 3**). Lab personnel were not blind to group assignment. Participants assigned to the training groups completed training sessions two to three times per week, for a total of 10 sessions. During each training session, four games were played in random order, with each game played for ~20 min each. After training was completed for the training groups or, after a comparable amount of time had elapsed for the no-contact control group, participants completed the same testing sessions in reverse session order.

**Table 2:** Transfer tasks.

Transfer tasks	Category	Order	Session	Reference
Shipley abstraction	Reasoning/gF	6	1	Zachary and Shipley (1986)
Paper folding	Reasoning/gF	8	1	Ekstrom etal. (1976)
Spatial relations	Reasoning/gF	10	1	Bennett etal. (1997)
Form boards	Reasoning/gF	11	1	Ekstrom etal. (1976)
Letter sets	Reasoning/gF	12	1	Ekstrom etal. (1976)
Matrix reasoning	Reasoning/gF	22	MRI	Ravens (1962), Crone etal. (2009)
Digit symbol substitution	Perceptual speed	1	1	Wechsler (1997a)
Pattern comparison	Perceptual speed	3	1	Salthouse and Babcock (1991)
Letter comparison	Perceptual speed	4	1	Salthouse and Babcock (1991)
Word recall	Episodic memory	2	1	Wechsler (1997b)
Logical memory	Episodic memory	5	1	Wechsler (1997b)
Paired associates	Episodic memory	9	1	Salthouse etal. (1996)
Visual short-term memory	Working memory	13	2	Luck and Vogel (1997)
Symmetry span	Working memory	16	2	Redick etal. (2012)
N-back	Working memory	17	3	Kirchner (1958), Kane etal. (2007)
Running span	Working memory	19	3	Broadway and Engle (2010)
Spatial working memory	Working memory	20	3	Erickson etal. (2011)
Trail making	Attention	7	1	Reitan (1958)
Attentional blink	Attention	14	2	Raymond etal. (1992)
Task switching	Attention	15	2	Kramer etal. (1999), Pashler (2000)
Color stroop	Attention	18	3	Stroop (1935), Stroop (1992)
Attention network test	Attention	21	MRI	Fan etal. (2002)
Bloxorz*	Game - reasoning/gF	23	MRI	miniclip.com
Dodge*	Game - attention	24	MRI	armorgames.com

All tasks were administered before and after the training sessions. *For these tasks, the original game developers created local MRI-compatible versions for the study.

**Table 3:** Training games.

Training games	Group	Description	Primary measure	Source
Silversphere	WM-REAS 1, WM-REAS 2	Move a sphere to a blue vortex by creating a path with blocks of different features, while avoiding falling off the platform and other obstacles.	Maximum level	miniclip.com
Digital Switch	WM-REAS 1	In the main game, switch “digibot” positions to collect falling coins corresponding to the same “digibot” color.	Maximum level	miniclip.com
TwoThree	WM-REAS 1	Shoot down rapidly presented numbers by pointing to them and subtracting the presented numbers down to 0 using units of 2 or 3.	Maximum level	armorgames.com
Sushi Go Round	WM-REAS 1	Serve a certain number of customers in the allotted time by learning and preparing different recipes correctly, cleaning tables, and ordering ingredients.	Maximum money earned	miniclip.com
Aengie Quest	WM-REAS 2	Get character (Aengie) to move across the board and exit each level by pushing switches and boxes, finding keys, and opening doors.	Maximum level	freegamesjungle.com
Gude Balls	WM-REAS 2	Explode all plates by filling a plate with four of the same colored balls and switching balls to other plates. Obstacles are introduced and combined in each level.	Maximum level	bigfishgames.com
Block Drop	WM-REAS 2	Move around a gem on three-dimensional blocks to remove all blocks except the checkered block. Unique block arrangements are presented in each level.	Maximum level	miniclip.com
Alphattack	Active control	Prevent bombs from landing by quickly typing the characters specified on the approaching bombs. There are three main stages of difficulty with levels in each.	Estimated maximum level (level~×~difficulty)	miniclip.com
Crashdown	Active control	Prevent the wall from reaching the top of the screen by clicking on groups of three or more same colored blocks.	Maximum level	miniclip.com
Music Catch 2	Active control	Earn points by mousing over streams of colored shapes and avoiding contiguously appearing red shapes.	Mean points	reflexive.com
Enigmata	Active control	Navigate a ship while avoiding and destroying enemies, and collecting objects that provide armor or power.	Maximum level	maxgames.com

*
Games performed by each training group along with the primary measure used for analyses.*

### COGNITIVE ASSESSMENT

Assessments administered before and after training were grouped into five categories: perceptual speed, reasoning/fluid intelligence (gF), working memory, episodic memory, and attentional control (selective visual attention, divided attention). Additionally, participants played two casual video games (one reasoning, one attention) that were not used as training games in any of the groups. Participants also completed the Behavior Rating Inventory of Executive Function Adult Version (Roth et al., 2005). Below is a brief description of each task, with more details in **Table 2**. At the very last testing session, participants were asked about study expectations and gaming experience in more detail. If participants reported in this post-experiment questionnaire that they played the testing or training games outside the laboratory, or were active video game players, their data was discarded from all the analyses. If a participant had 0% accuracy (except for Attentional Blink), a negative d-prime score (where applicable), or scored more than four standard deviations below the mean in a task (mean and standard deviation taken separately for each session), their data was excluded from training-related analyses of that task only. If the outlier data identified using the aforementioned methods was from the post-testing session, that participant’s pre-testing score was still used in the pre-test PCA.

#### Reasoning, episodic memory, and perceptual speed

With the exception of matrix reasoning, all tasks for these three constructs were taken from the Virginia Cognitive Aging Project (Salthouse and Ferrer-Caja, 2003; Salthouse, 2004, 2005, 2010). These tasks have been extensively and uniformly used so only brief descriptions are provided below.

***Word recall***. Participants listen to lists of words and recall the words in any order.

***Logical memory***. Participants listen to stories and recall the stories in detail.

***Paired associates***. Participants remember word pairs and recall the second word in the pair.

***Digit-symbol coding***. Participants write the corresponding symbol for each digit using a coding table for reference.

***Letter comparison and pattern comparison***. Participants determine whether a pair of patterns or letter combinations are the same or different.

***Form boards***. Participants choose shapes that will exactly fill a certain space.

***Spatial relations***. Participants identify the three-dimensional object that would match a folded two-dimensional object.

***Paper folding***. Participants identify the resulting pattern of holes from a sequence of folds and a punch through the folded sheet.

***Shipley abstract***. Participants identify the missing stimuli in a progressive sequence of letters, words, or numbers.

***Letter sets***. Participants see five patterns and identify the pattern that does not match the others.

***Matrix reasoning***. The Raven’s Progressive Matrices task was modified for a functional MRI paradigm and was largely based on a relational reasoning task used in Crone et al. (2009). Participants viewed a 3 × 3 matrix containing patterns in all but one cell and chose the best pattern out of three options to identify the missing piece. They solved two types of problems: control trials in which no integration was required across rows and columns, and reasoning trials that required integration of information across cells.

#### Working memory

***Visual short-term memory***. An array of four shapes briefly appeared on the screen. After a delay, a shape appeared and participants had to decide whether this stimulus was in the original array. The experiment consisted of three blocks with targets varying in color, shape, and conjunctions of color and shape in each block, respectively.

***N-back***. Participants viewed a sequence of centrally presented letters. For each letter, participants were instructed to determine if the letter was the same as the previous letter (first block), the same as the letter two back (second block), or the same as the letter three back (third block).

***Spatial working memory***. On each trial, a configuration of two, three, or four black dots was presented on the screen. After a brief delay, a red dot appeared and participants were instructed to determine if the red dot was in the same position as one of the black dots presented earlier in that trial.

***Running span***. Participants are presented a sequence of letters and are instructed to remember the last n items presented.

***Symmetry span***. Participants performed symmetry judgments while remembering a sequence of red squares within a matrix. Participants were asked to recall the order and locations of the previously presented sequence.

#### Attentional control

***Task switching***. Participants were asked to determine whether a number was odd or even, or whether it was higher or lower than five. The background color (blue or pink) determined the task to be performed. Participants completed two single task blocks and then a mixed task block where the task varied unpredictably across trials.

***Attentional blink***. Participants viewed sequences of rapidly presented black letters. In each sequence, a white letter appeared (location in sequence varied between trials) and on 50% of trials, a black “X” followed the white letter at varying lags. During the critical condition, participants were asked to identify the white letter and whether or not an X was presented.

***Trail making***. Participants first connected numbers distributed across a sheet of paper by drawing a line between numbers in ascending order. Participants then connected numbers and letters in alternating and ascending order on a second sheet.

***Attention network test***. Participants responded to the direction of a central arrow that pointed in the same (congruent) or opposite direction (incongruent) as four other adjacent arrows (two on each side). On some trials, warning cues appeared at the center of screen or at the location of the upcoming arrows. The task was adapted for the MRI environment, following procedures detailed in Fan et al. (2002).

***Color stroop***. Participants viewed a sequence of words and were asked to determine the color of the word. Three trial types were randomly presented: congruent (e.g., word “red” in red ink), neutral (e.g., word “dog” in red ink), or incongruent (e.g., word “red” in blue ink).

#### Casual video games used for assessment

***Dodge***. Participants aim to avoid enemy missiles that are actively chasing the ship under their control. Participants earn points and pass levels by guiding missiles into enemies.

***Bloxorz***. Participants rotate and move a rectangular block around a maze while avoiding falling off the platform. Levels are passed when the block reaches a target hole on the maze.

#### Self-report instruments

***Behavior rating inventory of executive function by PAR^**TM**^***. Participants indicated the frequency that they experienced a variety of executive function problems (never, sometimes, or often). The questionnaire included several dimensions: Inhibit, Shift, Emotional Control, Self-Monitor, Initiate, Working Memory, Plan/Organize, Organization of Materials, and Task Monitor.

***Post-experiment questionnaire***. Participants completed a form that inquired about gameplay and lifestyle history as well as their experience in the study. In one section (hereafter referred to as perceived improvement questions), they were asked to rate whether they felt that participation in the study changed the following functions: overall intelligence, short-term or working memory, long-term memory, ability to pay attention or focus, ability to pay attention to multiple things at once (divided attention), hand-eye or visuomotor coordination, perception, vision or visual acuity, problem-solving ability, multi-tasking ability, reasoning ability, academic performance, spatial visualization ability, emotional regulation, and productivity at work or school, or tendency to procrastinate. Participants were also asked to give feedback and elaborate on strategies used in the training games, report whether they played any assessment or training games outside the lab (with no penalty to their participation in the study), and answer other questions on the nature of their knowledge and experience with video games.

### CASUAL GAMES USED FOR TRAINING

The WM-REAS 1 training group was formed using games that were highly correlated with performance on working memory and reasoning tasks, and the active control training group was composed of games that were not highly correlated with working memory and reasoning tasks (Baniqued et al., 2013). After about 20 participants were run in each of these two groups, we included an adaptive reasoning training (WM-REAS 2) group and a no-contact control group. The WM-REAS 2 group played games that also showed high and comparable correlations (as the WM-REAS 1 games) with working memory and reasoning tasks^3^. Unlike the first two training groups where adaptiveness in three out of the four games was only *within session* (exceptions: Silversphere in WM-REAS 1 and Alphattack in active control), participants in the WM-REAS 2 group started on the level that they ended on in the previous session, such that the games were adaptive *across* sessions. Games were embedded and played on a research portal designed for the study by Digital Artefacts^4^. **Table 3** contains brief descriptions of each game played by the groups. After the first, fifth, and last training sessions, training participants were asked to answer the following questions for each game, rating their answers on a scale of 1–10 (1 = least, 5 = neutral, 10 = greatest): (1) How much did you enjoy/like each game, (2) How engaging was each game, (3) How demanding/effortful was each game, and (4) How motivated were you to achieve the highest possible score on each game?

## RESULTS

We first analyze the training games to determine practice-related improvement across the 10 sessions of training. We also assess whether the training groups differed in their experience with their respective games. In the next section, we determine whether game training transfers to untrained tasks by comparing performance on the pre- and post-assessment tasks, first at a construct-level and then at the individual task-level to determine the consistency of the effects. Since transfer to untrained tasks may vary depending on initial cognitive ability, we also investigated the effect of baseline fluid intelligence (reasoning) ability on transfer effects. We then examined whether perceived improvement in cognitive abilities differs across the training groups, which would prompt a re-analysis of the transfer affects to take into account expectations. Finally, we analyze other variables that may affect the effectiveness of training.

### PRACTICE EFFECTS

#### Game performance across sessions

**FIGURE 1:**
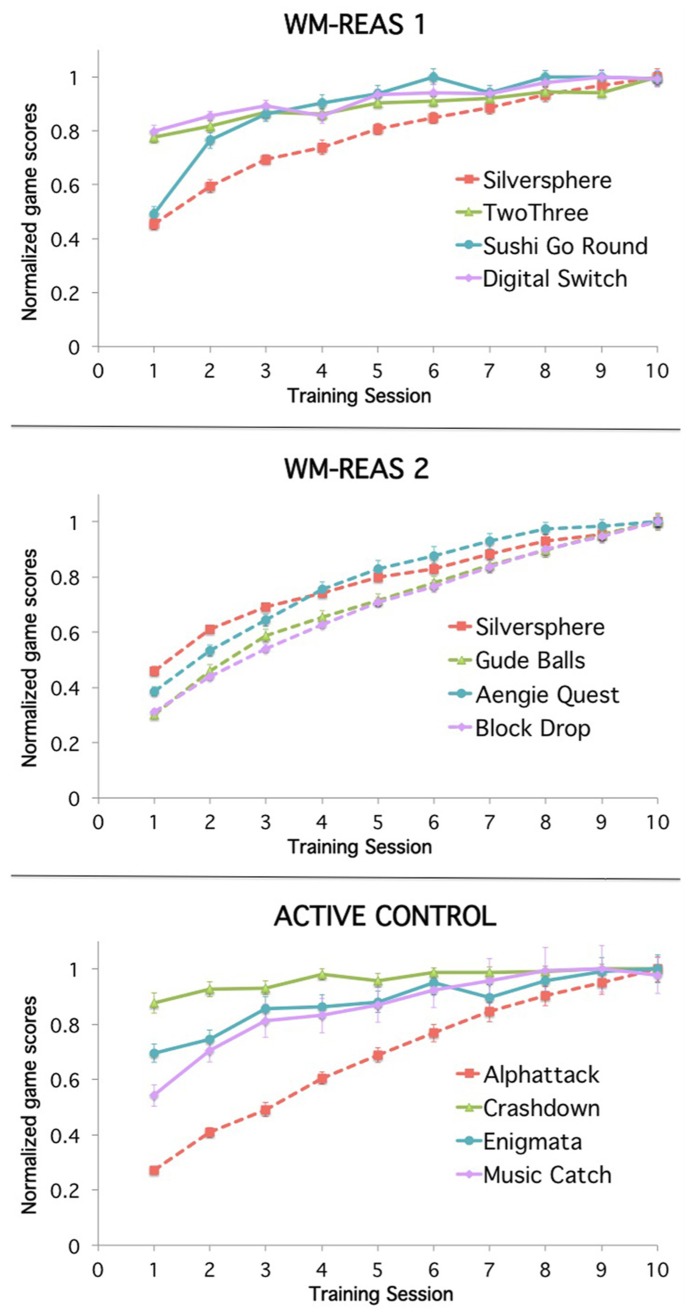
**Mean training game performance as a function of group and session.** Group average scores at each session, normalized by each game’s maximum average score: WM-REAS 1: Silversphere = 18.861, TwoThree = 20.265, Sushi-Go-Round = 5785.429, Digital Switch = 8.161; WM-REAS 2: Silversphere = 19.421, Gude Balls = 14.474, Aengie Quest = 20.526, Block Drop = 52.385; Active Control: Alphattack = 51.867, Music Catch = 4032358.24, Crashdown = 6.667, Enigmata = 4.069. Dashed lines indicate games that were adaptive across sessions. Error bars represent ±SEM.

All groups improved on their respective training games, regardless of whether the games were adaptive across sessions. If participants completed the last level of any across-session adaptive game, they started back at level one. For analysis purposes, the data for these succeeding sessions was replaced with the maximum score or level. Repeated measures ANOVA with session as a within-subjects factor (10 time points) was conducted for the primary measure of each game. The practice effects were robust, with significant main effects of session at *p* < 0.001 for all games. In games like Sushi Go Round, where participants started at level one at each session and thus highest level completed plateaued over time, participants improved in other aspects of the game such as in total number of customers served. Group averages are plotted in **Figure 1**, with scores divided by the maximum average score of each game for ease of presentation.

#### Game experience across sessions

**FIGURE 2:**
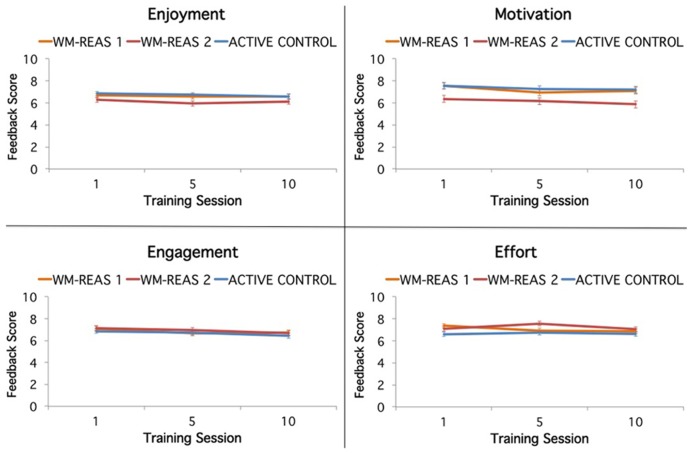
**Training game feedback as a function of group and session.** Feedback regarding game enjoyment, motivation, engagement, and effort were collected during the first, fifth, and last training sessions. Feedback scale: 1 = least, 5 = neutral, 10 = greatest. Error bars represent ±SEM.

The four feedback questions of enjoyment, engagement, motivation, and effort were entered separately into repeated measures ANOVAs with group as between-subjects factor and time (training sessions 1, 5, and 10) as within-subjects factor. Ratings for each question were averaged across the four games played by each participant. Results are summarized in **Figure 2**.

For enjoyment, there was no group × time interaction, and no main effects of group and time. For engagement, there was no main effect of group, and no group × time interaction, but a main effect of time where engagement decreased across sessions [*F*(2,216) = 7.389, *p* = 0.001, $\eta_{p}^{2}$ = 0.064]. For motivation, there was no group × time interaction, but a main effect of time [*F*(2,222) = 5.026, *p* = 0.007, $\eta_{p}^{2}$ =0.043] with decreased motivation over sessions, and a main effect of group [*F*(2,111) = 6.035, *p* = 0.003, $\eta_{p}^{2}$ = 0.098], with lower motivation for the WM-REAS 2 group compared to the WM-REAS 1 and active control groups (*p*s < 0.05). For effort, there was no main effect of time, but a main effect of group [*F*(2,111) = 3.339, *p* = 0.045, $\eta_{p}^{2}$ = 0.054], where effort ratings were higher for the WM-REAS 2 group compared to the active control group (*p* = 0.017). The WM-REAS groups were not different from each other and the WM-REAS 1 group did not differ from the active control group. The group × time interaction was significant [*F*(4,222) = 2.913, *p* = 0.022, $\eta_{p}^{2}$ = 0.050], with effort ratings for WM-REAS 2 peaking at the fifth session compared to the first session peak for WM-REAS 1. When only taking into account the first and last session, the group × time interaction was not significant [*F*(2,115) = 2.364, *p* = 0.099, $\eta_{p}^{2}$ = 0.039]. Overall, the feedback questions indicated that the three training groups were comparable in their experience of the games, although the WM-REAS 2 group reported lower motivation overall and higher effort but only at mid-training, likely due to the greater demand from the adaptive games.

Qualitative feedback regarding strategies and overall experience for each game can be found at .

### TRANSFER OF TRAINING

#### Composite-level analyses

To ascertain whether game training had any general effect on cognitive abilities and to better address the issue of measurement error and multiple comparisons, we performed analyses at the construct level using composite scores derived by averaging standardized improvement scores (post-test – pre-test/standard deviation of pre-test, collapsed across groups) from related tasks. These task groupings were confirmed by a PCA on the pre-test data. Despite the smaller sample size (*n* = 116, using all subjects with baseline data of each task) and the addition of several measures, the PCA was comparable with the previous validation study (Baniqued et al., 2013), with seven interpretable components that in combination explained 57% of the variance (**Table 4**): reasoning or fluid intelligence (Matrix Reasoning, Paper Folding, Form Boards, Spatial Relations, Letter Sets, Shipley Abstract, Bloxorz), perceptual speed (Digit Symbol, Pattern Comparison, Letter Comparison), episodic memory (Word Recall, Logical Memory, Paired Associates), ANT-visual attention (ANT alerting, orienting effects), divided attention (Dodge, Attention Blink, Trail Making), and two working memory components [N-back, Spatial WM, Visual short-term memory (STM), Running Span, Symmetry Span], with a notable separation between more simple (Component 6: Spatial WM, N-back, Visual STM) and complex (Component 7: Symmetry Span, Running Span) working memory tasks. We also reran the PCA without the ANT measures and the results were similar, with interpretable components of fluid intelligence, perceptual speed, episodic memory, divided attention, and working memory.

**Table 4:** Transfer tasks: principal components analysis using baseline data.

PCA of transfer tasks (pre-test scores only)	Component									
	1	2	3	4	5	6	7	8	9	10
Spatial relations	0.828									
Form boards	0.727									
Paper folding	0.682									
Shipley abstraction	0.574		0.337							
Letter sets	0.564									-0.533
Matrix reasoning	0.563									
Spatial STM	0.506					0.418			-0.33	
Pattern comparison		0.794								
Digit symbol coding		0.764								
Letter comparison		0.735								
Symmetry span	0.402	0.515					0.399			
Word recall			0.8							
Paired associates			0.787							
Logical memory			0.633					0.351		
ANT alerting				0.803						
ANT orienting				-0.714				0.345		
Dodge					0.764					
N-back	0.464				0.529	0.428				
Attentional blink				0.385	-0.512					0.34
Trail making					-0.427		-0.427	-0.404		
Task switch local cost						-0.699				
Visual STM	0.307			-0.365		0.611				
Running span							0.81			
ANT conflict								0.75		
Stroop									0.908	
Bloxorz	0.406									0.711

*Standardized component loadings from PCA solution, showing components with eigenvalues greater than 1. For clarity, only loadings above 0.30 are displayed. Rotation method: varimax with Kaiser normalization. Listwise exclusion was performed (n = 116).*

Because of the smaller PCA sample size and for ease of interpretation, only tasks that were consistent with previous literature were included in the component score calculations (e.g., WM measures that loaded highly onto the first component were excluded from the gF composite score). Given the overlap of simple and complex WM measures in Components 1, 6, and 7, we combined the simple and complex WM measures into one composite score.

**FIGURE 3:**
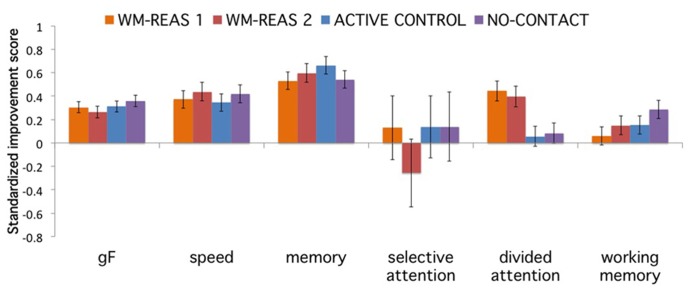
**Transfer gain as a function of composite and group.** Error bars represent ±SEM.

We conducted ANOVAs on the composite gain scores with group as a between-subjects factor and found a significant group effect for divided attention [*F*(3,166) = 5.613, *p* = 0.001, $\eta_{p}^{2}$ = 0.092], with higher gain scores for both WM-REAS training groups (**Figure 3**). No group effects were found for fluid intelligence [*F*(3,166) = 0.667, *p* = 0.573, $\eta_{p}^{2}$ = 0.012], perceptual speed [*F*(3,166) = 0.316, *p* = 0.814, $\eta_{p}^{2}$ = 0.006], episodic memory [*F*(3,166) = 0.637, *p* = 0.592, $\eta_{p}^{2}$ = 0.011], ANT-visual attention [*F*(3,154) = 0.468, *p* = 0.705, $\eta_{p}^{2}$ = 0.009] and working memory [*F*(3,166) = 1.388, *p* = 0.248, $\eta_{p}^{2}$ = 0.024].

ANOVAs on composite scores that included all tasks with loadings of greater than 0.30 yielded similar results. The ANT composite also yielded a non-significant result when the alerting and orienting effects were summed with equal positive weight. The results were also similar for a re-analysis without the no-contact control group; training effects were only found in divided attention [*F*(2,124) = 6.676, *p* = 0.002, $\eta_{p}^{2}$ = 0.097].

#### Task-level analyses

To check whether the groups performed equivalently at pre-testing, one-way ANOVAs with group as between-subjects factor (all four groups) were conducted for all pre-test primary measures reported in **Table 5**. At baseline, group differences were only found in Trail Making measures (*p* = 0.039 for Trails B–A, *p* = 0.063 for Trail B). None of the other measures differed among groups at pre-testing (*p*s > 0.13).

**Table 5:** Mean task performance as a function of training group and session.

Transfer results				WM-REAS 1		WM = REAS 2		Active control		No-contact	
Task	Measure	Group (4) × session	Group (3) × session	Pre	Post	Pre	Post	Pre	Post	Pre	Post
Digit Symbol	Total correct	*F*(3,163) = 0.739, *p* = 0.530, $\eta_{p}^{2}$ = 0.013	*F*(2,122) = 1.072, *p* = 0.346, $\eta_{p}^{2}$ = 0.017	93.881 (2.123)	99.69 (1.979)	91.525 (2.175)	99.775 (2.028)	96.093 (2.098)	103.093 (1.956)	94.786 (2.123)	101.762 (1.979)
Pattern Comparison	Mean correct	*F*(3,166) = 1.065, *p* = 0.366, $\eta_{p}^{2}$ = 0.019	*F*(2,124) = 1.629, *p* = 0.200, $\eta_{p}^{2}$ = 0.026	21.465 (0.529)	23.442 (0.5)	20.575 (0.549)	22.225 (0.518)	21.682 (0.523)	22.716 (0.494)	21.651 (0.529)	23.291 (0.5)
Letter Comparison	Mean correct	*F*(3,166) = 0.370, *p* = 0.775, $\eta_{p}^{2}$ = 0.007	*F*(2,124) = 0.304, *p* = 0.739, $\eta_{p}^{2}$ = 0.005	13.302 (0.359)	13.57 (0.397)	12.50 (0.373)	13.062 (0.412)	12.977 (0.355)	13.523 (0.392)	13.058 (0.359)	13.779 (0.397)
Logical Memory	Total correct	*F*(3,166) = 0.252, *p* = 0.860, $\eta_{p}^{2}$ = 0.005	*F*(2,124) = 0.090, *p* = 0.914, $\eta_{p}^{2}$ = 0.001	48.535 (1.373)	52.721 (1.353)	48.325 (1.423)	52.575 (1.403)	48.227 (1.357)	52.977 (1.338)	49.14 (1.373)	52.581 (1.353)
Word Recall	Total correct	*F*(3,166) = 1.275, *p* = 0.285, $\eta_{p}^{2}$ = 0.023	*F*(2,123) = 0.862, *p* = 0.425, $\eta_{p}^{2}$ = 0.014	53.651 (0.939)	58.512 (0.86)	52.692 (0.986)	58.974 (0.903)	53.341 (0.928)	58.636 (0.85)	54.302 (0.939)	58.884 (0.86)
Paired Associates	Accuracy	*F*(3,160) = 1.105, *p* = 0.349, $\eta_{p}^{2}$ = 0.020	*F*(2,118) = 1.360, *p* = 0.261, $\eta_{p}^{2}$ = 0.023	0.618 (0.039)	0.701 (0.035)	0.716 (0.04)	0.791 (0.035)	0.661 (0.039)	0.807 (0.035)	0.678 (0.038)	0.8 (0.034)
Form Boards	Total correct	*F*(3,165) = 0.462, *p* = 0.709, $\eta_{p}^{2}$ = 0.008	*F*(2,123) = 0.249, *p* = 0.780, $\eta_{p}^{2}$ = 0.004	9.628 (0.624)	12.326 (0.642)	9.897 (0.656)	12.872 (0.674)	10.909 (0.617)	13.364 (0.634)	10.14 (0.624)	12.302 (0.642)
Paper Folding	Total correct	*F*(3,166) = 0.775, *p* = 0.509, $\eta_{p}^{2}$ = 0.014	*F*(2,124) = 0.587, *p* = 0.557, $\eta_{p}^{2}$ = 0.009	7.953 (0.343)	8.698 (0.301)	8.8 (0.355)	9.3 (0.312)	8.932 (0.339)	9.773 (0.297)	8.279 (0.343)	9.256 (0.301)
Spatial Relations	Total correct	*F*(3,164) = 0.232, *p* = 0.874, $\eta_{p}^{2}$ = 0.004	*F*(2,123) = 0.191, *p* = 0.827, $\eta_{p}^{2}$ = 0.003	11.535 (0.675)	13.023 (0.623)	12.1 (0.7)	13.25 (0.646)	13.395 (0.675)	14.674 (0.623)	12.452 (0.683)	14 (0.63)
Letter Sets	Total correct	*F*(3,162) = 0.271, *p* = 0.846, $\eta_{p}^{2}$ = 0.005	*F*(2,121) = 0.333, *p* = 0.718, $\eta_{p}^{2}$ = 0.005	12.095 (0.252)	12.571 (0.249)	12.564 (0.261)	12.744 (0.258)	12.884 (0.249)	13.14 (0.246)	12.381 (0.252)	12.786 (0.249)
Shipley Abstract	Total correct	*F*(3,166) = 0.163, *p* = 0.921, $\eta_{p}^{2}$ = 0.003	*F*(2,124) = 0.197, *p* = 0.821, $\eta_{p}^{2}$ = 0.003	15.814 (0.326)	16.86 (0.294)	15.325 (0.338)	16.575 (0.305)	15.909 (0.322)	17.114 (0.291)	15.419 (0.326)	16.674 (0.294)
Matrix Reasoning	Reasoning accuracy	*F*(3,164) = 0.559, *p* = 0.643, $\eta_{p}^{2}$ = 0.010	*F*(2,124) = 0.629, *p* = 0.535, $\eta_{p}^{2}$ = 0.010	79.302 (1.456)	73.411 (1.513)	78.667 (1.509)	71.25 (1.569)	77.689 (1.439)	72.955 (1.496)	78.211 (1.491)	73.415 (1.55)
	Control accuracy	*F*(3,164) = 0.080, *p* = 0.971, $\eta_{p}^{2}$ = 0.001	*F*(2,124) = 0.106, *p* = 0.900, $\eta_{p}^{2}$ = 0.002	97.519 (0.55)	97.597 (0.652)	97.083 (0.57)	96.667 (0.676)	97.803 (0.544)	97.727 (0.644)	97.48 (0.563)	97.48 (0.668)
Bloxorz	Last level completed	*F*(3,158) = 0.422, *p* = 0.738, $\eta_{p}^{2}$ = 0.008	*F*(2,119) = 0.094, *p* = 0.910, $\eta_{p}^{2}$ = 0.002	4.317 (0.153)	4.976 (0.153)	4.41 (0.157)	5.154 (0.157)	4.405 (0.151)	5.119 (0.151)	4.35 (0.155)	5.225 (0.155)
Dodge	Last level completed	*F*(3,159) = 3.199, *p* = 0.025, $\eta_{p}^{2}$ = 0.057	*F*(2,118) = 2.192, *p* = 0.116, $\eta_{p}^{2}$ = 0.036	8.897 (0.144)	9.538 (0.109)	8.925 (0.142)	9.45 (0.107)	8.929 (0.139)	9.167 (0.105)	9.095 (0.139)	9.167 (0.105)
Attentional Blink	Lag 8 – lag 2 accuracy	*F*(3,164) = 4.327, *p* = 0.006, $\eta_{p}^{2}$ = 0.073	*F*(2,124) = 5.085, *p* = 0.008, $\eta_{p}^{2}$ = 0.076	0.345 (0.045)	0.298 (0.045)	0.39 (0.046)	0.269 (0.047)	0.347 (0.044)	0.402 (0.044)	0.469 (0.046)	0.358 (0.046)
	Lag 2 accuracy	*F*(3,164) = 1.616, *p* = 0.118, $\eta_{p}^{2}$ = 0.029	*F*(2,124) = 1.749, *p* = 0.178, $\eta_{p}^{2}$ = 0.027	0.424 (0.039)	0.504 (0.044)	0.367 (0.04)	0.482 (0.045)	0.444 (0.038)	0.475 (0.043)	0.359 (0.04)	0.482 (0.045)
	Lag 8 accuracy	*F*(3,164) = 2.656, *p* = 0.050, $\eta_{p}^{2}$ = 0.046	*F*(2,124) = 3.407, *p* = 0.036, $\eta_{p}^{2}$ = 0.052	0.769 (0.026)	0.802 (0.024)	0.757 (0.027)	0.751 (0.025)	0.792 (0.026)	0.876 (0.024)	0.828 (0.027)	0.84 (0.025)
Trail Making	Trails B–A RT (s)	*F*(3,161) = 4.271, *p* = 0.006, $\eta_{p}^{2}$ = 0.074	*F*(2,119) = 2.090, *p* = 0.128, $\eta_{p}^{2}$ = 0.0341	26.958 (1.699)	20.863 (1.593)	25.746 (1.81)	23.232 (1.697)	21.241 (1.679)	20.146 (1.574)	21.657 (1.679)	24.489 (1.574)
	Trails A (s)	*F*(3,161) = 0.258, *p* = 0.856, $\eta_{p}^{2}$ = 0.005	*F*(2,119) = 0.007, *p* = 0.993, $\eta_{p}^{2}$ < 0.001	27.592 (1.423)	23.434 (0.995)	25.608 (1.516)	21.527 (1.061)	26.497 (1.406)	22.222 (0.984)	26.306 (1.406)	20.962 (0.984)
	Trails B (s)	*F*(3,161) = 3.596, *p* = 0.015, $\eta_{p}^{2}$ = 0.063	*F*(2,119) = 2.255, *p* = 0.109, $\eta_{p}^{2}$ = 0.037	54.549 (2.053)	44.297 (1.864)	51.354 (2.187)	44.759 (1.986)	47.737 (2.029)	42.368 (1.842)	47.963 (2.029)	45.451 (1.842)
Task Switching	Switch-repeat RT (ms)	*F*(3,159) = 0.539, *p* = 0.656, $\eta_{p}^{2}$ = 0.010	*F*(2,118) = 0.722, *p* = 0.488, $\eta_{p}^{2}$ = 0.012	267.731 (16.745)	259.396 (18.525)	290.985 (17.393)	272.458 (19.242)	246.059 (16.544)	258.306 (18.303)	274.598 (16.544)	276.566 (18.303)
	Switch-repeat accuracy	*F*(3,159) = 0.729, *p* = 0.536, $\eta_{p}^{2}$ = 0.014	*F*(2,118) = 0.177, *p* = 0.889, $\eta_{p}^{2}$ = 0.002	-0.021 (0.007)	-0.016 (0.008)	-0.019 (0.007)	-0.019 (0.009)	-0.015 (0.007)	-0.015 (0.008)	-0.016 (0.007)	-0.029 (0.008)
	Single-repeat RT (ms)	*F*(3,159) = 0.689, *p* = 0.560, $\eta_{p}^{2}$ = 0.013	*F*(2,118) = 0.714, *p* = 0.492, $\eta_{p}^{2}$ = 0.012	192.3 (15.638)	172.87 (14.56)	202.926 (16.243)	168.306 (15.123)	180.445 (15.45)	180.937 (14.385)	206.949 (15.45)	173.492 (14.385)
	Single-repeat accuracy	*F*(3,159) = 0.661, *p* = 0.577, $\eta_{p}^{2}$ = 0.012	*F*(2,118) = 0.956, *p* = 0.387, $\eta_{p}^{2}$ = 0.016	0.023 (0.012)	0.032 (0.011)	0.031 (0.012)	0.019 (0.012)	0.014 (0.012)	0.029 (0.011)	0.023 (0.012)	0.021 (0.011)
Attention Network Test	No cue – center, RT (ms)	*F*(3,152) = 0.063, *p* = 0.979, $\eta_{p}^{2}$ = 0.001	*F*(2,116) = 0.079, *p* = 0.924, $\eta_{p}^{2}$ = 0.001	21.315 (5.42)	23.341 (6.287)	18.001 (5.866)	24.66 (6.805)	14.248 (5.292)	20.26 (6.139)	18.173 (5.705)	24.184 (6.618)
	Location – center, RT (ms)	*F*(3,152) = 0.192, *p* = 0.902, $\eta_{p}^{2}$ = 0.004	*F*(2,116) = 0.057, *p* = 0.945, $\eta_{p}^{2}$ = 0.001	63.479 (6.695)	54.724 (7.472)	50.722 (7.246)	45.086 (8.087)	58.953 (6.537)	54.6 (7.296)	54.19 (7.047)	55.472 (7.865)
	Inc-con, RT (ms)	*F*(3,152) = 0.672, *p* = 0.571, $\eta_{p}^{2}$ = 0.013	*F*(2,116) = 0.759, *p* = 0.471, $\eta_{p}^{2}$ = 0.013	132.402 (9.33)	117.847 (8.849)	134.627 (10.099)	112.92 (9.578)	125.825 (9.111)	120.688 (8.641)	122.882 (9.822)	103.643 (9.315)
Stroop	Inc-neu, RT (ms)	*F*(3,162) = 1.019, *p* = 0.386, $\eta_{p}^{2}$ = 0.019	*F*(2,122) = 1.395, *p* = 0.252, $\eta_{p}^{2}$ = 0.022	51.439 (8.095)	45.966 (7.404)	47.529 (8.196)	53.566 (7.496)	62.07 (7.814)	47.242 (7.147)	59.814 (8.095)	59.616 (7.404)
	Inc-con, RT (ms)	*F*(3,162) = 0.100, *p* = 0.960, $\eta_{p}^{2}$ = 0.002	*F(*2,122) = 0.115, *p* = 0.891, $\eta_{p}^{2}$ = 0.002	82.642 (8.795)	78.122 (8.166)	87.682 (8.904)	82.264 (8.268)	80.306 (8.49)	80.088 (7.883)	85.58 (8.795)	85.25 (8.166)
Running Span	Total correct	*F*(3,163) = 1.847, *p* = 0.141, $\eta_{p}^{2}$ = 0.033	*F*(2,122) = 2.414, *p* = 0.094, $\eta_{p}^{2}$ = 0.038	21.833 (0.885)	21.952 (0.959)	22.2 (0.906)	21.525 (0.983)	20.721 (0.874)	22.814 (0.948)	21.19 (0.885)	22.738 (0.959)
Symmetry Span	Total correct	*F*(3,123) = 0.797, *p* = 0.498, $\eta_{p}^{2}$ = 0.019	*F*(2,83) = 1.129, *p* = 0.328, $\eta_{p}^{2}$ = 0.026	18.19 (1.811)	20.476 (1.972)	19.55 (1.312)	24.65 (1.429)	21.08 (1.659)	25.56 (1.807)	18.22 (1.296)	22.756 (1.411)
Spatial STM	Accuracy	*F*(3,164) = 2.436, *p* = 0.067, $\eta_{p}^{2}$ = 0.043	*F*(2,122) = 1.103, *p* = 0.149, $\eta_{p}^{2}$ = 0.031	0.882 (0.013)	0.852 (0.015)	0.862 (0.013)	0.853 (0.015)	0.883 (0.012)	0.892 (0.014)	0.856 (0.012)	0.878 (0.014)
	d′	*F*(3,164) = 0.822, *p* = 0.484, $\eta_{p}^{2}$ = 0.015	*F*(2,122) = 1.178, *p* = 0.311, $\eta_{p}^{2}$ = 0.019	2.941 (0.157)	2.807 (0.199)	2.641 (0.159)	2.634 (0.202)	2.846 (0.152)	3.065 (0.192)	2.789 (0.153)	2.95 (0.195)
Visual STM	Overall accuracy	*F*(3,165) = 0.443, *p* = 0.722, $\eta_{p}^{2}$ = 0.008	*F*(2,123) = 0.443, *p* = 0.643, $\eta_{p}^{2}$ = 0.007	0.795 (0.009)	0.795 (0.009)	0.804 (0.009)	0.795 (0.01)	0.812 (0.009)	0.806 (0.009)	0.795 (0.009)	0.795 (0.009)
	Overall d′	*F*(3,165) = 0.455, *p* = 0.714, $\eta_{p}^{2}$ = 0.008	*F*(2,123) = 0.502, *p* = 0.606, $\eta_{p}^{2}$ = 0.008	1.741 (0.065)	1.761 (0.07)	1.784 (0.068)	1.721 (0.072)	1.84 (0.065)	1.813 (0.07)	1.737 (0.065)	1.748 (0.07)
	Both d′	*F*(3,165) = 0.388, *p* = 0.761, $\eta_{p}^{2}$ = 0.007	*F*(2,123) = 0.324, *p* = 0.724, $\eta_{p}^{2}$ = 0.006	1.308 (0.11)	1.411 (0.105)	1.327 (0.114)	1.315 (0.109)	1.281 (0.11)	1.384 (0.105)	1.287 (0.11)	1.254 (0.105)
	Color d′	*F*(3,165) = 1.316, *p* = 0.271, $\eta_{p}^{2}$ = 0.023	*F*(2,123) = 1.249, *p* = 0.290, $\eta_{p}^{2}$ = 0.020	3.029 (0.182)	2.757 (0.192)	2.815 (0.188)	3.019 (0.199)	2.943 (0.182)	3.008 (0.192)	2.9 (0.182)	2.638 (0.192)
	Shape d′	*F*(3,165) = 0.357, *p* = 0.784, $\eta_{p}^{2}$ = 0.006	*F*(2,123) = 0.423, *p* = 0.656, $\eta_{p}^{2}$ = 0.007	1.857 (0.159)	1.887 (0.129)	1.842 (0.165)	1.674 (0.134)	1.979 (0.159)	1.884 (0.129)	1.999 (0.159)	1.803 (0.129)
N-back	2-Back d′	*F*(3,161) = 0.287, *p* = 0.835, $\eta_{p}^{2}$ = 0.005	*F*(2,120) = 0.432, *p* = 0.650, $\eta_{p}^{2}$ = 0.007	4.551 (0.361)	4.59 (0.355)	4.07 (0.38)	4.153 (0.373)	4.723 (0.357)	5.261 (0.351)	4.338 (0.361)	4.535 (0.355)
	3-Back d′	*F*(3,161) = 2.072, *p* = 0.106, $\eta_{p}^{2}$ = 0.037	*F*(2,120) = 2.049, *p* = 0.133, $\eta_{p}^{2}$ = 0.033	2.354 (0.219)	2.672 (0.26)	2.228 (0.23)	2.869 (0.273)	2.589 (0.216)	2.454 (0.257)	2.002 (0.219)	2.597 (0.26)
	2-Back accuracy	*F*(3,161) = 0.753, *p* = 0.522, $\eta_{p}^{2}$ = 0.014	*F*(2,120) = 0.923, *p* = 0.400, $\eta_{p}^{2}$ = 0.015	0.947 (0.007)	0.951 (0.009)	0.938 (0.008)	0.931 (0.009)	0.955 (0.007)	0.964 (0.009)	0.947 (0.007)	0.955 (0.009)
	3-Back accuracy	*F*(3,161) = 2.008, *p* = 0.115, $\eta_{p}^{2}$ = 0.036	*F*(2,120) = 1.271, *p* = 0.284, $\eta_{p}^{2}$ = 0.021	0.882 (0.012)	0.88 (0.012)	0.85 (0.013)	0.878 (0.013)	0.861 (0.012)	0.878 (0.012)	0.845 (0.012)	0.885 (0.012)

*
ANOVA results are shown for analyses with and without the no-contact control group. Pre-subtraction data are only displayed for tasks where the difference or effect score was significant. Parentheses indicate ±SEM.*

To evaluate transfer of training, repeated measures ANOVAs were performed for each task, with time as a within-subjects factor and group as a between-subjects factor. The ANOVAs were rerun without the no-contact control group and the results were similar, although the effects described below were less robust and at times no longer significant at *p* < 0.05. For brevity, results for analyses with and without the no-contact control group are shown in **Table 5**.

Significant group × time interactions at *p* < 0.05 were found in Dodge, Attentional Blink and Trail-Making, which were also the three tasks that made up the divided attention composite. *Post hoc* tests revealed that both WM-REAS groups reached higher levels of Dodge at post-test (time effect *p* < 0.001 for both groups), while only the WM-REAS 1 group showed a reduced Trails cost at post-test (*p* < 0.01).

Because the Trail-Making measures had significant group differences at baseline, driven by longer Trail B times for WM-REAS 1 and WM-REAS 2, we excluded the pre and post-testing data of subjects with the two longest baseline times in each WM-REAS group (which were also the four highest times overall across all groups) so that group mean values were comparable at baseline. These data points were not outliers as identified by methods described earlier. One-way ANOVAs on the subset of data confirmed that the groups were no longer significantly different at baseline. After excluding the longest times, the results were similar to the analysis with all subjects (**Table 5**), with the Trails B–A group × time interaction still significant at [*F*(2,126) = 3.373, *p* = 0.020, $\eta_{p}^{2}$ = 0.061].

The magnitude of the attentional blink was smaller at post-test for the WM-REAS 2 (*p* < 0.001) and no-contact control (*p* < 0.01) groups. Since the pattern of results is complex^5^, we also analyze lag 2 and lag 8 separately. The group by time interaction for lag 8 was driven by increased performance at post-test for the active control group (*p* < 0.001). For lag 2, the time effect was significant for the no-contact control (*p* = 0.002), WM-REAS 1 (*p* = 0.026) and WM-REAS 2 (*p* < 0.001) groups. Taken together, the results for lag 2, lag 8, and the difference effect (lag 8 – lag 2) suggest that the reduced blink effect is only reliable in the WM-REAS 2 group.

#### Baseline reasoning ability and transfer: composite-level analysis

To determine whether training may be more or selectively effective for those with lower abilities at initial testing, we correlated transfer gains with baseline reasoning or gF ability (pre-training composite of Matrix Reasoning, Paper Folding, Form Boards, Spatial Relations, Letter Sets, Shipley Abstract), which provides an estimate of general mental ability (Gray and Thompson, 2004).

Pre-training gF correlated with gains in divided attention, such that participants with lower baseline gF had larger gains from training. This was significant only for the WM-REAS 1 group (*r* = -0.327, *p* = 0.032) and the WM-REAS 2 group (*r* = -0.333, *p* = 0.036).

An ANCOVA on the divided attention gain composite with the three training groups as between-subjects factor and with baseline gF as a covariate revealed a significant effect of training after controlling for baseline gF [*F*(2,123) = 5.509, *p* = 0.005, $\eta_{p}^{2}$ = 0.082], with larger gains from the WM-REAS groups. Baseline gF was confirmed to have an effect on divided attention gain [*F*(1,123) = 6.113, *p* = 0.015, $\eta_{p}^{2}$ = 0.047]. To confirm lack of transfer in other abilities, ANCOVAs with baseline gF as a covariate were also conducted on the other composites. The findings were consistent with previous analyses as no group effects were found.

To test the robustness of the divided attention gains in the WM-REAS groups, we reran the composite-level ANCOVAs after excluding the highest performers (upper quartile) in each group and still found a significant group effect in divided attention (and not in other cognitive abilities), with higher gains in the WM-REAS groups. This was true in analyses with [*F*(3,124) = 5.554, *p* = 0.001, $\eta_{p}^{2}$ = 0.118) and without the no-contact control group [*F*(2,92) = 6.199, *p* = 0.003, $\eta_{p}^{2}$ = 0.119].

Pre-training gF also correlated with gains in reasoning for the WM-REAS 1 (*r* = -0.320, *p* = 0.036), active control (*r* = -0.299, *p* = 049), and no-contact control (*r* = -0.440, *p* = 0.003) groups. Pre-training gF also correlated with perceptual speed (*r* = 0.360, *p* = 0.018), but this was only true for the WM-REAS 1 group.

### PERCEIVED IMPROVEMENT

#### Post-experiment survey

Compared to the no-contact control group (12.5%), a greater percentage of participants in the three training groups reported that the study changed the way they performed their daily activities, “in a good way” [χ^2^(3) = 10.010, *p* = 0.018, WM-REAS 1 = 33.3%, WM-REAS 2 = 43.6%, active control = 37.2%)]. There was no difference between training groups when the no-contact control group was excluded from the chi-square analysis [χ^2^(2) = 0.917, *p* = 0.639]. Due to the low frequency of responses in the “Yes, but not in a good way” category (WM-REAS 1 = 2, WM-REAS 2 = 1, active control = 1, no-contact = 0), we excluded this option in the chi-square tests.

All groups reported that their overall skill at videogames was higher at post-test [*F*(1,164) = 217.620, *p* < 0.001, $\eta_{p}^{2}$ = 0.570], but a group × session interaction [*F*(3,164) = 4.802, *p* = 0.003, $\eta_{p}^{2}$ = 0.081] revealed that the training groups rated themselves significantly higher than the no-contact control group. There was, however, no difference between the three training groups in perceived video game skill after training [*F*(2,125) = 0.070, *p* = 0.933, $\eta_{p}^{2}$ = 0.001].

**FIGURE 4:**
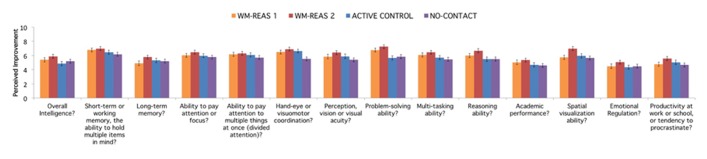
**Perceived improvement as a function of group.** Average responses for each group, including data from WM-REAS 1 and active control participants who did not receive a web-based version of the post-experiment survey. Error bars represent ±SEM.

Due to experimenter error that resulted in a change in instructions when a web-based form of the survey was administered, for the perceived improvement questions, we only present statistics for subjects who received the same electronic version of the post-experiment survey (although all subjects are included in **Figure 4** to provide the general pattern of results). In the initial written survey completed by 22 out of 44 subjects in WM-REAS 1, and 16 out of 44 subjects in the active control group, participants checked a box to indicate whether the study changed that particular ability, and then rated the extent of the change (1 = very poorly, 10 = very desirably). In the web-based survey, each item required an answer. That is, participants had to rate change on the ability on a scale of 1–10, which lent more ambiguity as an answer of 1 could now be interpreted as no change or negative change.

Separate question ANOVAs revealed a significant group effect at *p* < 0.05 for working memory [*F*(3,126) = 2.765, *p* = 0.045], hand-eye or visuomotor coordination [*F*(3,126) = 5.332, *p* = 0.002], multitasking [*F*(3,126) = 6.714, *p* < 0.001], problem-solving [*F*(3,126) = 2.944, *p* = 0.036], reasoning [*F*(3,126) = 3.730, *p* = 0.013], and academic performance [*F*(3,126) = 4.530, *p* = 0.005], with higher ratings in general for the training groups compared to the no-contact control group. When the perceived improvement questions were analyzed without the no-contact control group, however, only the group effects for multitasking [*F*(2,88) = 6.300, *p* = 0.003] and academic performance [*F*(2,87) = 3.305, *p* = 0.041] remained significant, although none of the *post hoc* comparisons between groups were significant at *p* < 0.05.

#### Behavioral rating inventory of executive function

Repeated measures ANOVA revealed a significant group × time interaction only for the Shift index (problems transitioning between activity, strategy or situation), both when the no-contact control group was included in the analyses [*F*(3,141) = 3.995, *p* = 0.009, $\eta_{p}^{2}$ = 0.078], and when it was not [*F*(2,94) = 5.129, *p* = 0.008, $\eta_{p}^{2}$ = 0.098]. Paired *t*-tests revealed that this was due to an increase in Shift problems for the WM-REAS 1 group, although this effect must be taken lightly as the WM-REAS 1 group also had a lower mean Shift score at pre-test compared to the other groups, and only 21 subjects in this group completed the questionnaire at both time-points. Given the limited range of answers (never, sometimes, often) and the relatively weak task effects, it is possible that the BRIEF questionnaire could not adequately measure any subtle changes or differences between groups. Overall, the BRIEF results are consistent with the perceived improvement findings where majority of participants reported little to no improvement in cognitive functions or daily activities.

### EXPLORATORY ANALYSIS: OTHER INDIVIDUAL DIFFERENCES AND TRANSFER GAIN

We found that initial reasoning/gF ability predicted gains in divided attention, so we went a step further and conducted an exploratory analysis of other individual differences that may influence the effectiveness of WM-REAS casual game training. A few studies have found that training-related transfer is predicted by the amount of improvement in the trained tasks, such that greater “responders” show greater transfer (Jaeggi et al., 2011, 2013). We examined this in the current study by correlating transfer gain composite scores with training gain composite score. For each individual in each training group, we calculated the difference between performance in the later sessions (9, 10) and performance in the early sessions (1, 2). This difference score was then divided by the standard deviation in the early sessions (within each group). Standardized scores for the four games were then averaged to form a training gain composite score. Correlations conducted separately for each training group did not reveal any significant relationship between training gain and transfer gains, even after controlling for baseline game performance.

Mixed results from previous studies, coupled with small sample sizes and population demographic differences suggest the contribution of other factors such as gender, motivation, and other pre-existing abilities to training effectiveness (Jaeggi et al., 2013). Thus, for the WM-REAS groups, correlations were conducted between each transfer gain composite score and the following factors: gender, game play habits (only <3 h/week; combined modalities), training game experience (enjoyment, engagement, motivation, and effort after fifth and last training sessions), bilingualism, exercise (Godin Leisure-Time questionnaire), time spent watching television/movies, sleeping, reading books/magazines/newspapers, surfing the web, on social network sites, meditating, in nature, learning a new language, and learning a new instrument. Given the within-group and exploratory nature of this analysis, we only state correlations that were significant at *p* < 0.01.

For the WM-REAS 1 group, more time on social network sites (*r* = 0.458, *p* = 0.002) correlated with higher divided attention gains, and more time spent reading correlated with gains in fluid intelligence (*r* = 0.461, *p* = 0.002).

For the WM-REAS 2 group, game effort at mid-training correlated with gains in divided attention (*r* = 0.443, *p* = 0.008) such that greater effort was associated with larger gains. There was also correlation between sleep and gains in ANT-visual attention gain (*r* = 0.470, *p* = 0.004).

We did not find significant correlations with the other factors, which may be due to the lack of variability or lack of representation in certain conditions (e.g., maximum of less than 3 h weekly video game play), especially given the predominantly collegiate make-up of the sample.

## DISCUSSION

We examined whether widely available casual video games can broadly improve cognition by demonstrating transfer to untrained tasks. In our relatively sizeable sample (approximately 40 participants in each group), we found that while participants improved on trained games, transfer to untrained tasks was limited. Playing casual video games for 15 h did not improve most aspects of cognition, but playing working memory and reasoning casual games improved divided attention, with some caveats to be noted. As several of the training tasks involve working memory and reasoning demands in several fast-paced situations, and given our findings of higher divided attention gains for those with lower initial reasoning ability, we also provide a link between the working memory and action video game training literature.

### EFFECTS OF WM-REAS TRAINING ON COGNITIVE FUNCTION

Groups trained on working memory and reasoning games improved in a composite measure of divided attention. All three tasks used for the divided attention score (Dodge, Attentional Blink, Trail Making) involve paying attention to multiple targets, with little demand on maintaining internal representations of stimuli. Multi-object tracking demands were also part of the active control games (Enigmata, Alphattack, Crashdown, MusicCatch), but it is likely that the lack of reasoning or planning demands in the games led to a more passive strategy as participants only reacted to objects as they appeared on the screen. Indeed, participant feedback for the active control games contained more statements about psychomotor strategies such as clicking as quickly as possible in response to stimuli. On the other hand, the WM-REAS groups practiced a mix of speeded and non-speeded tasks, with the speeded tasks (Silversphere, Sushi-Go-Round, DigiSwitch, TwoThree, Gude Balls) requiring both planning ahead and attention to multiple stimuli on the screen. The additional management demands in the WM-REAS games may have better developed divided attention skills as coordinated execution of multiple elements was critical to success in many games.

In the initial game validation study (Baniqued et al., 2013), fluid intelligence best predicted performance on multi-object tracking games such as Dodge, with Dodge performance also significantly correlating with performance on reasoning games (and not just attention or multiple-object tracking games). These findings can be taken as evidence of near transfer when taking into account the previously demonstrated relationship between Dodge and reasoning ability, and relatively far transfer given the dissimilar surface features of the trained games and transfer tasks such as Dodge. Such transfer to untrained paradigms bolsters the idea that the complex and more externally valid environment found in strategy-heavy video games may provide a more useful and practical platform for developing cognitive skills (Green et al., 2010).

These results are consistent with findings that playing strategy-demanding time-limited games can enhance attention skills (Green and Bavelier, 2003, 2006a,b, 2007; Basak et al., 2008; Hubert-Wallander et al., 2011a; Glass et al., 2013; Oei and Patterson, 2013). More strikingly, our findings parallel research (Bavelier et al., 2012) showing that active video game players perform better in a variety of attention-demanding tasks, including the attention blink and multiple-object tracking paradigms. We did not find improvements in the Attention Network Test, but this is not entirely unexpected in the context of other findings that active video game players do not show benefits for exogenous attention (Hubert-Wallander et al., 2011b). It is especially interesting that despite also playing fast-paced and attention-demanding games, the active control group did not improve to the level of the participants who practiced games with greater reasoning and working memory demands.

Working memory capacity has repeatedly been shown to correlate with attention abilities, with findings that capacity can predict the magnitude of the attentional blink (Arnell and Stubitz, 2010). We did not find increases in working memory capacity or fluid intelligence, but it is plausible that such changes in higher-level abilities evolve more slowly than changes in lower level attention abilities, following the developmental trajectory of processing speed, working memory, and fluid intelligence (Fry and Hale, 1996; Kail, 2007; Coyle et al., 2011). Alternatively, it may be that at least in young adults, training abilities such as working memory does not improve capacity *per se*, but more lower-level attention or information processing mechanisms that overlap or are common elements across reasoning, working memory, and other attentional control paradigms (Thorndike, 1913). In fact, Kundu et al. (2013) found that while dual n-back training did not improve fluid intelligence or complex working memory span, training improved “efficiency of stimulus processing”, as indexed by improvements in visual search and short-term memory. More and more studies find that training on a single adaptive working memory task does not transfer to working memory capacity or fluid intelligence (Chooi and Thompson, 2012; Redick et al., 2012; Lilienthal et al., 2013; Thompson et al., 2013), and studies that do find transfer observe them in attention measures (Chein and Morrison, 2010; Kundu et al., 2013; Oelhafen et al., 2013). On the other hand, it is also worth mentioning that a greater variety of training tasks may be more effective for demonstrating transfer to higher-level abilities. A study that trained participants on multiple working memory tasks for an average of 30 h over 12 weeks resulted in gains in several measures of reasoning, although the sample size in this study was relatively small (Jauvs, 2012), and transfer to other cognitive domains such as attention was not assessed. While the pattern of transfer results depends on the nature of the training tasks, overall the evidence points to working memory training as weakly beneficial for fluid intelligence, but promising in terms of enhancing attention skills.

A common difficulty in intervention studies is employing appropriate control groups to address placebo effects. We attempted to overcome this here by using multiple training groups and measuring performance expectations after training. Despite all training groups reporting equivalent increases in perceived videogame skill, only the reasoning groups improved in Dodge performance. This is especially interesting given that the active control games emphasized processing speed and tracking multiple objects on the screen. We found a group effect in multi-tasking expectations, however, the pairwise comparisons between training groups was not significant. Moreover, training feedback showed that the groups were generally comparable in enjoyment, engagement, motivation and effort. The WM-REAS 2 group reported less motivation overall and slightly greater effort at mid-training, which is likely due to the greater demands from the across-session adaptive games. Such reported challenge or difficulty can be argued to account for the transfer results, though this does not explain why the WM-REAS 1 group also demonstrated transfer even without differences in perceived effort or motivation during training. It is likely that in the context of this experiment where individuals are paid for simply playing games, motivation does not play a significant role in determining training effectiveness.

Although we cannot determine whether only a subset of WM-REAS games led to the effects in the reasoning groups, we can infer that playing a variety of reasoning games promoted more generalizable skills as opposed to task mastery. Taatgen (2013) makes a compelling argument that tasks such as working memory and task switching promote development of “proactive” control that encourages endogenous preparation. As several of the WM-REAS games and strategy video games involve fast-paced decision making, endogenous preparation likely comes into play such that sequence of actions are planned ahead of time and deployed quickly at the right moment. Conversely, it can be argued that the active control games promoted more “reactive” control that is not measurable in the cognitive abilities we tested. Taatgen further makes the argument that executive function training improves “skill” and not “capacity,” which echoes a sentiment articulated by Luck and Vogel (2013) that greater working memory capacity may not lead to better problem-solving, but that individuals who can flexibly develop strategies to enhance performance may more ably execute working memory and other related tasks (Kirby and Lawson, 1983). Participants in the WM-REAS groups completed a variety of challenges in the WM-REAS games and practiced these problem solving skills (with many self-reports of “trying out new combinations, strategies”) under demanding and in some occasions, extremely time-limited conditions. This idea of enhanced decision-making under high load is also a main explanation provided for why playing fast-paced action games leads to improvement in attention-demanding tasks (Hubert-Wallander et al., 2011a; Mishra et al., 2011). In this regard, our findings are in line with previous research and extend the literature by showing that game-related improvement in attention skills may also result from non-violent gaming environments.

This study was conducted with healthy young adults, which limits the extension of these results to other populations. However, the correlation between divided attention transfer gain and baseline reasoning, selected as a proxy for general ability (Gray and Thompson, 2004), suggests that these kinds of protocols may be more useful in populations that have more to gain from training, such as children or older adults who experience age-related cognitive decline. This relationship between pre-existing differences in cognitive ability and training efficacy also offers an explanation for the mixed results in training studies. As most working memory training studies have relatively small sample sizes (for a review, see Morrison and Chein, 2011), individual differences may enhance or obscure any effects of training on a subset of participants.

### LIMITATIONS AND FUTURE DIRECTIONS

We acknowledge that other factors such as improvement expectations may influence the transfer results. However, due to the ambiguity of the scale in the perceived improvement questions, we could not reliably factor in expectations in the statistical analyses. Nonetheless, it is interesting to note that the training groups did not significantly differ in perceived improvement, and that the WM-REAS groups improved in divided attention, an ability where their expectations did not differ from the active control group. Although we found a group difference in perceived multitasking improvement, which can be taken as related to divided attention, the *post hoc* comparisons were not significant. Moreover, no improvements were found in Task Switching or in Symmetry Span, both of which clearly involved managing multiple tasks.

It should also be noted that the divided attention composite includes tasks that are not as extensively evaluated as the tasks used to estimate reasoning, perceptual speed and episodic memory abilities. Nonetheless, similarities in Dodge, Attention Blink and Trail Making were confirmed by a PCA and give us more confidence in the divided attention score. We also revisited the validation study and found correlations between Dodge, Attention Blink and Trail-Making measures. The tasks may also be sensitive to practice effects, although all groups performed the same tests and no improvements were found in the control groups. Nonetheless, this training approach needs to be re-examined with a more extensive battery of attentional control tasks to shed light on why benefits were not observed in tasks like Symmetry Span which also involved divided attention, albeit in the form of shifting from one task to another. The tasks that showed transfer involved distributing attention across objects in space (Trail Making, Dodge), or across a narrow time frame, as is the case with Attentional Blink, but this needs to be further evaluated.

It can also be argued that the improvement in the WM-REAS groups was due to a change in strategy when performing the assessments. This is worthwhile to explore in future studies since working memory–reasoning tasks may not improve divided attention *per se*, but planning or reasoning abilities that may be best observed or manifested in such “divided attention” tasks. It may also be the case that despite their high correlations with working memory and reasoning, the WM-REAS games demanded other skills for successful gameplay over the course of training, with a shift of emphasis from reasoning to divided attention skills as participants gained mastery of the games. Indeed, the degree to which reasoning ability predicts performance has been shown to change, with declining influence at later points of skill acquisition (Ackerman, 1988; Quiroga et al., 2009, 2011).

Ceiling performance and practice effects due to lack of alternate versions for six out of the seven fluid intelligence tasks (including Bloxorz) may contribute to the null effect in fluid intelligence, although note that gains were also not observed in the matrix reasoning task used in the magnet, which presented unique items at pre- and post-testing, with lower post-testing performance overall due to task design (**Table 5**). This null finding is consistent with decades-old literature showing that fluid intelligence is relatively stable in adulthood (Jensen, 1969; though with age-related decreases) and further challenges the claim that cognitive training can lead to improvement in this ability (Jaeggi et al., 2008; Sternberg, 2008). However, it is conceivable that the game training period in the current study was too short to train such an ability, and that more hours of practice may result in stronger and broader effects on cognition. Some participants also reached ceiling performance in the training games, so it would be useful to test whether playing more demanding games can lead to transfer to higher-level abilities of working memory and reasoning. In a recent experiment, Glass et al. (2013) found increases in cognitive flexibility following 40 h of real-time strategy game play (StarCraft) that emphasized a variety of skills including reasoning, working memory, and rapid switching in an adaptive and integrated setting.

Real-world measures of divided attention are needed to verify whether playing working memory and reasoning casual games can transfer to useful skills in daily life. Moreover, we did not conduct follow-up retention tests, so it is not known whether benefits persist beyond the training period. It is to be expected, however, in the same way as physical exercise, that continued practice is essential to maintaining or reaping intervention-related benefits.

Other interventions have been shown to improve cognition, and we provide modest evidence that playing casual games is *one possible means* to improve attention skills. The relatively non-violent nature of casual games compared to first-person shooter games also minimizes concerns regarding the negative effects of video game play. Nevertheless, with the aggressive marketing of brain games and the liberal application of preliminary training results, we caution against using video games or other computer-based programs as a sole or primary approach to improving brain function, particularly if it leads to a more sedentary lifestyle or in the words of Weis and Cerankosky (2010) “displace(s) activities that might have greater educational value.” Activities such as physical exercise have repeatedly been shown to benefit not only overall physical health, but also neurocognitive function (Hillman et al., 2008; Voss et al., 2013). Future studies should investigate the effects of combined and synergistic interventions to elucidate the ways in which activities may commonly and differentially change brain function. The goal of this line of research is not simply to evaluate the efficacy of interventions or the superiority of one over another, but to identify several avenues that promote a better quality of life, as a program that works for a certain population may not be suitable for another.

## AUTHOR CONTRIBUTIONS

All the authors contributed to designing the study. Pauline L. Baniqued and Michael B. Kranz supervised data collection and analyzed the data. Pauline L. Baniqued wrote the first draft of the manuscript with help from Michael B. Kranz for the section “Materials and Methods.” All the authors revised the manuscript.

## Conflict of Interest Statement

The authors declare that the research was conducted in the absence of any commercial or financial relationships that could be construed as a potential conflict of interest.

## Footnotes

1 For an example, see

2

3

4

5
