# Corrigendum: Cognitive training with casual video games: points to consider

**DOI:** 10.3389/fpsyg.2014.00234

**Published:** 2014-03-20

**Authors:** Pauline L. Baniqued, Michael B. Kranz, Michelle W. Voss, Hyunkyu Lee, Joshua D. Cosman, Joan Severson, Arthur F. Kramer

**Affiliations:** ^1^Department of Psychology, Beckman Institute for Advanced Science and Technology, University of Illinois at Urbana Champaign Urbana, IL, USA; ^2^Department of Psychology, University of Iowa Iowa City, IA, USA; ^3^Brain Plasticity Institute San Francisco, CA, USA; ^4^Department of Psychology, Vanderbilt University Nashville, TN, USA; ^5^Digital Artefacts, LLC Iowa City, IA, USA

**Keywords:** attention, working memory, reasoning, fluid intelligence, video games, cognitive training, casual games, transfer of training

The original publication contained an error that does not impact the significant findings and does not invalidate any conclusions derived from the study. In the WM-REAS 2 group, we inadvertently included data from one subject whose performance in the Attention Network Test (ANT) during post-testing met the exclusionary criteria. This resulted in an exaggerated negative transfer effect for the WM-REAS 2 group. After excluding this subject, the WM-REAS ANT-selective attention (also ANT-visual attention in the original manuscript) data is comparable with the other training groups (Figure 1). The results are consistent after reanalysis, with no significant transfer effect in ANT-selective attention [*F*_(3, 154)_ = 0.004, *p* = 1.000, η^2^_*p*_ < 0.001]. The reported association between sleep and ANT-selective attention in the original publication is no longer significant (*r* = 0.177, *p* = 0.310). The authors deeply regret this error.

**Figure 1:**
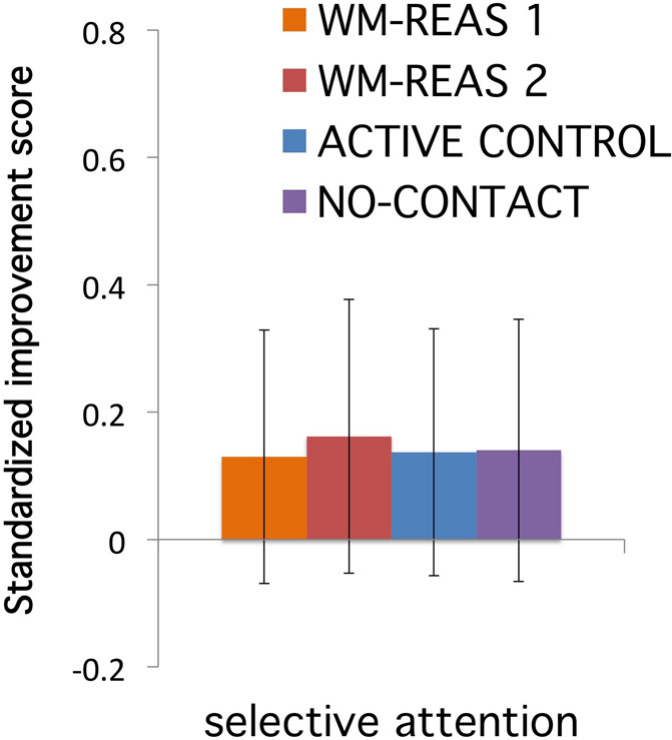
**Transfer effects for divided attention**.

